# Supplementary material for: Tuning instability in suspended monolayer 2D materials
Source: Nat Commun. 2024 May 13;15:4033. doi: 10.1038/s41467-024-48345-7 (PMC11091077; doi:10.1038/s41467-024-48345-7)
Supplement: Supplementary file 1 — Supplementary Information [file 41467_2024_48345_MOESM1_ESM.docx]

Supplementary Materials for

**Tuning Instability in Suspended Monolayer 2D Materials**

Yuan Hou^1,2,#^, Jingzhuo Zhou^2,#^, Zezhou He^3,#^, Juzheng Chen^2^, Mengya Zhu^2^, Hengan Wu^3^, Yang Lu^1^*

^1^Department of Mechanical Engineering, The University of Hong Kong, Pokfulam 999077, Hong Kong SAR, China

^2^Department of Mechanical Engineering, City University of Hong Kong, Kowloon 999077, Hong Kong SAR, China

^3^CAS Key Laboratory of Mechanical Behavior and Design of Materials, Department of Modern Mechanics, CAS Center for Excellence in Complex System Mechanics, University of Science and Technology of China, Hefei 230027, China

^#^These authors contributed equally: Yuan Hou, Jingzhuo Zhou, Zezhou He.

*Corresponding author: Yang Lu (ylu1@hku.hk)

**This PDF file includes:**

Supplementary Text

Supplementary Figs. 1 to 19

Supplementary References

**Supplementary Fig. 1 | Optical images of single-crystalline monolayer graphene and MoS_2_.** **a,** Monolayer graphene were exfoliated from bulk flake graphite. **b,** Monolayer MoS_2_ were prepared by CVD growth: MoO_3_ (99.999% purity) was utilized as a source of molybdenum and solid sulfur (99.999% purity) was used as a source of sulfur. Argon gas was used as a growth carrier gas in the MoS_2_ growth technique. During growth, MoO_3_ was heated to 650 degrees and sulfur was heated to 180 degrees. Here, the growth air pressure was 5000 Pa, and the growth took place for 5 minutes in an 80 mm pipe diameter dual-temperature zone tube furnace. The triangular single-crystalline monolayer MoS_2_ flakes were grown on a silicon wafer with a 300 nm oxide layer.

**Supplementary Fig. 2 | Raman spectra of monolayer graphene and MoS_2_.** **a,** The Raman spectra of monolayer graphene. **b,** The Raman spectra of monolayer MoS_2_. Both Raman characterization results showed that the monolayer 2D materials were free of defects and residual strain.

**Supplementary Fig. 3 | Schematic of transfer process.** **a-c,** Graphene flakes were exfoliated onto a piece of Si wafer with a 300 nm oxide layer (pre-treated by O_2_ plasma to remove contamination and increase surface adhesion) using the scotch tape, after which the monolayer sheets were selected based on optical contrast examination and checked by Raman spectroscopy. A wedging transfer method^1, 2^ was adapted to transfer the chosen graphene flake onto the micro-mechanical device (MMD). A thin PMMA stamp (prepared by spin coating) was attached to the flake through hot press as the supporting layer, and the combination could be released from the Si substrate by simply wedging in DI water. Next, the PMMA/graphene stack was transferred onto the MMD and precisely positioned under the optical microscope by a micromanipulator probe equipped with a tungsten needle. A thin water layer between the stack and the MMD was preserved during the alignment to ensure smooth movement and avoid material damage due to direct contact. The graphene flake was deposited onto the desired gap of the MMD once the water layer evaporated, and the capillary effect provides robust attachment between the material and substrate, followed by an 80℃ annealing which further enhances the adhesion. The final suspended graphene device was obtained after the removal of PMMA supporting layer using acetone and a critical point drying process. The MoS_2_ device was fabricated through the same process.

**Supplementary Fig. 4 |** **Comparison of relative displacements between substrate and graphene layers before and after shear loading.** **a-b,** The SEM images of the sample before and after shear loading, respectively. As shown in Supplementary Fig. 4**a**, three impurity particles immobilized on the upper right, lower right, and lower left regions on the substrate were labeled to be Markers 1, 2, and 3, respectively. The distances of these markers from their nearest neighboring graphene edges are 656 nm, 273 nm, and 938 nm, respectively. The horizontal distance between Marker 1 and Marker 2 is 742 nm. When the bottom Si substrate was displaced horizontally relative to upper Si substrate through shear loading, the distance between Markers 1 and 2 was measured as 992 nm. By comparing Supplementary Figs. 4**a** and **b**, it can be seen that only the distances between Markers 1 and 2 changed, while the distances between the three markers and their nearest graphene edges remains invariant. Secondly, the key parameter controlling the boundary slip is the interfacial shear resistance between 2D material and silicon substrate. Previous studies have shown that the shear resistance between 2D material and silicon substrate is about 1~2 MPa, which is much higher than that between 2D materials, and thus it can be regarded as a strong interface^3, 4, 5^. In addition, since the surface of PTS is not atomically smooth during the preparation process, defects and morphological undulations on the silicon substrate surface can also have the pinning effect on the 2D material. Here, we estimate for the possibility of sliding of monolayer graphene on the substrate. According to previous work^6^, the critical sliding stress can be described by the shear-lag model, given by $\sigma_{s}=\tau_{cr}L/t$, where *L* is the overlap length, *t* is the graphene thickness (~ 0.34 nm). The shear strength $\tau_{cr}$ between graphene and the substrate is about 1~2 MPa. Using the smallest overlap length in Supplementary Fig. 4**a** (*L* ~ 1.62 μm), the critical sliding stress *σ_s_* > 4.76 GPa, which is higher than the maximum principal stress during push-to-shear process. Consequently, the sliding of monolayer graphene on the substrate cannot occur in our testing samples.

**Supplementary Fig. 5 |** **Reversible instability of monolayer MoS_2_.** **a,** The SEM images of MoS_2_ during the wrinkling-smoothing process. **b,** The schematics of the wrinkling-smoothing process of MoS_2_. Analogous to graphene, MoS_2_ exhibits instability and recovery processes during shear loading and unloading: as shear strain increases, wrinkling evolve with decreasing wavelength. Once the strain is reduced, the wrinkling structure would be smoothed and eventually recover to their original state. Scale bars: **a** 1μm.

**Supplementary Fig. 6 |** **The original loading-unloading curve of the sample**. In our experiments, PTS MMD is fixed to a metal holder by a conductive carbon adhesive tape due to its inherent damping properties, which serve to mitigate the instrument-induced vibrations. Supplementary Fig. 6 shows the original force-displacement curves during loading-unloading, where the stress-strain curves of the sample can be extracted by subtract the stiffness of PTS. When PTS was loaded by diamond indenter pushing, a hysteresis in the force-displacement curve occurs at large strains because of the carbon adhesive tape has viscoelasticity. It should be noted that while the viscoelasticity of the adhesive tape can potentially influence stress measurements at larger strains, our emphasis in this study is on the evolution of shear strain versus wavelength. Therefore, the hysteresis curve in stress-strain relationship will not affect our analysis of instability behavior.

**Supplementary Fig. 7 |** **The orientation of wrinkles of graphene and MoS_2_.** Scale bars: 1 μm.

**Supplementary Fig. 8 |** **Splitting of shear-wrinkling.** **a-f,** The schematics show the in-plane shear model of 2D materials, $F$ is the shear force applied to material, $l$ is the displacement of the upper boundary and $\delta$ is the pretension displacement of suspended film, $L$ and $W$ is length and width of the suspended film, respectively; $\gamma$ is the shear strain of film; $\varepsilon_{pre}$ is pretension strain applied on film. $x \& y$ and $\omega\& \psi$ are the coordinates built on the initial square thin film and central wrinkling structure, respectively. The dashed line represents the wrinkling structure where $\beta$ is the angle between wrinkles and $x$ axis (From the direction of the principal stress, it can be inferred that the angle $\beta$ is about 45°). $\lambda$ is the half-wavelength of wrinkle and $z$ denotes the out-of-plane coordinate of thin film.

**Note 1**

**Shearing model:**

In the case of in-plane shearing, the stress and strain of thin film have the following relationship (several models were developed for thin film shearing^7, 8^):

$$\begin{aligned} \varepsilon_{x}=\frac{\partial u}{\partial x}=0; \varepsilon_{y}=\frac{\partial v}{\partial y}=\frac{\delta}{W}=\varepsilon_{pre}; \gamma_{xy}=\frac{\partial u}{\partial y}+\frac{\partial v}{\partial x}=\frac{l}{W}=\gamma\#\left( 1 \right) \end{aligned}$$

$$\begin{aligned} \sigma_{x}=\frac{Ev}{1-v^{2}}\varepsilon_{y}; \sigma_{y}=\frac{E}{1-v^{2}}\varepsilon_{y}; \tau_{xy}=\frac{E}{2\left( 1+v \right)}\gamma_{xy}\#\left( 2 \right) \end{aligned}$$

The principal stress and direction can be obtained as,

$$\begin{aligned} \sigma_{max; min}=\frac{E\delta}{2\left( 1-v \right)W}\pm\frac{E}{2\left( 1+v \right)}\sqrt{\left( \frac{\delta}{W} \right)^{2}+\left( \frac{l}{W} \right)^{2}}\#\left( 3 \right) \end{aligned}$$

Here, $\varepsilon_{pre}=\frac{\delta}{W}$ is the pretension strain; and $\gamma=\frac{l}{W}$ is the shear strain. Note that, the maximum and minimum principal stresses correspond to the stresses on the $\psi$ axis and $\omega$ axis of the wrinkling structure, respectively,

$$\sigma_{\omega}=\frac{E}{2(1-v)}\varepsilon_{pre}-\frac{E}{2\left( 1+v \right)}\sqrt{{\varepsilon_{pre}}^{2}+\left( \gamma\right)^{2}};$$

$$\begin{aligned} \sigma_{\psi}=\frac{E}{2\left( 1-v \right)}\varepsilon_{pre}+\frac{E}{2\left( 1+v \right)}\sqrt{{\varepsilon_{pre}}^{2}+\left( \gamma\right)^{2}}.\#\left( 4 \right) \end{aligned}$$

As shown in Supplementary Fig. 8, the shape function of wrinkling structure can be assumed as the product of the double sinusoidal functions.

$$\begin{aligned} z\left( \omega,\psi\right)=hsin\left( \frac{i\pi\omega}{\lambda} \right)sin\left( \frac{j\pi\psi sin\beta}{W} \right)\#\left( 5 \right) \end{aligned}$$

The bending energy of wrinkle is written as,

$$\begin{aligned} U_{b}=\frac{D}{2}\iint\left( ▽^{2}z \right)^{2}d\omega d\psi=\frac{\pi^{4}\lambda WD}{8sin\beta}h^{2}\left( \frac{i^{2}}{\lambda^{2}}+\frac{j^{2}{sin\beta}^{2}}{W^{2}} \right)^{2} \#\left( 6 \right) \end{aligned}$$

The stretch energy is written as,

$$U_{s}=\frac{1}{2}\iint[\sigma_{\omega}t\left( \frac{\partial z}{\partial\omega} \right)^{2}+\sigma_{\psi}t\left( \frac{\partial z}{\partial\psi} \right)^{2}+{2\tau}_{\omega\psi}t(\frac{\partial z}{\partial\omega}\frac{\partial z}{\partial\psi})]d\omega d\psi$$

$$\begin{aligned} =\frac{\pi^{2}Wi^{2}}{8\lambda sin\beta}\sigma_{\omega}th^{2}+\frac{\pi^{2}\lambda j^{2}sin\beta}{8W}\sigma_{\psi}th^{2}\#\left( 7 \right) \end{aligned}$$

Total energy of the wrinkle is,

$$\begin{aligned} {U=U}_{b}+U_{s}=\frac{\pi^{4}\lambda WD}{8sin\beta}A^{2}\left( \frac{i^{2}}{\lambda^{2}}+\frac{j^{2}{\sin\beta}^{2}}{W^{2}} \right)^{2}+\frac{\pi^{2}Wi^{2}}{8\lambda sin\beta}\sigma_{\omega}tA^{2}+\frac{\pi^{2}\lambda j^{2}sin\beta}{8W}\sigma_{\psi}tA^{2}\#\left( 8 \right) \end{aligned}$$

**Primary (1^st^) instability:**

The necessary condition for the appearance of the wrinkling structure is that the following formula has the non-zero solution,

$$\begin{aligned} \frac{\partial U}{\partial A}=0\#\left( 9 \right) \end{aligned}$$

Thus, the above problem is transformed into an eigenvalue problem,

$$\begin{aligned} \frac{\pi^{2}Wi^{2}}{8\lambda\sin\beta}\sigma_{\omega}t=-\frac{\pi^{4}\lambda WD}{8sin\beta}\left( \frac{i^{2}}{\lambda^{2}}+\frac{j^{2}{\sin\beta}^{2}}{W^{2}} \right)^{2}-\frac{\pi^{2}\lambda j^{2}\sin\beta}{8W}\sigma_{\psi}t\#\left( 10 \right) \end{aligned}$$

The critical stress can be solved as,

$$\begin{aligned} \sigma_{\omega}=-\pi^{2}D\left( \frac{i}{\lambda}+\frac{j^{2}\lambda{\sin\beta}^{2}}{iW^{2}} \right)^{2}-\frac{\lambda^{2}j^{2}{\sin\beta}^{2}}{W^{2}i^{2}}\sigma_{\psi}\#\left( 11 \right) \end{aligned}$$

For a primary instability problem, it corresponds to a wavenumber of 1 ($i=j=1$) in both directions, so that,

$$\begin{aligned} {\sigma_{\omega}|}_{i=1,j=1}\to-\pi^{2}D\left( \frac{1}{\lambda}+\frac{\lambda{\sin\beta}^{2}}{W^{2}} \right)^{2}-\frac{\lambda^{2}{\sin\beta}^{2}}{W^{2}}\sigma_{\psi}\#\left( 12 \right) \end{aligned}$$

Here, we recall the Eq. 1 to determine the $\sigma_{\psi}$. Since that $\sigma_{\omega}$ approaches to zero (consider that monolayer 2D materials has extremely low bending stiffness), we assume $\sigma_{\omega}\to0$ when the primary instability happens, then we can obtain (from Eq. 4) the critical shear strain as,

$$\begin{aligned} {\gamma^{cr1}|}_{\sigma_{\omega}\to0}\sim\frac{2\varepsilon_{pre}\sqrt{v}}{1-v}\#\left( 13 \right) \end{aligned}$$

Therefore, $\sigma_{\psi}$ can be written as,

$$\begin{aligned} {\sigma_{\psi}^{cr1}|}_{\sigma_{\omega}\to0}=\frac{E}{2\left( 1-v \right)}\varepsilon_{pre}+\frac{E}{2\left( 1+v \right)}\sqrt{{\varepsilon_{pre}}^{2}+\left( {\gamma^{cr1}|}_{\eta\to0} \right)^{2}}=\frac{E}{1-v}\varepsilon_{pre}\#\left( 14 \right) \end{aligned}$$

Plugging Eq. 15 into Eq. 13, we can get that,

$$\begin{aligned} {\sigma_{\omega}|}_{i=1,j=1}=-\pi^{2}D\left( \frac{1}{\lambda}+\frac{\lambda{\sin\beta}^{2}}{W^{2}} \right)^{2}-\frac{\lambda^{2}{\sin\beta}^{2}}{W^{2}}\frac{E}{1-v}\varepsilon_{pre}\#\left( 15 \right) \end{aligned}$$

Considering that $\lambda\ll W^{2}$, the critical stress for primary instability can be simplified as,

$$\begin{aligned} {\sigma_{\omega}|}_{i=1,j=1}\sim-\left( \frac{\pi^{2}D}{\lambda^{2}t}+\frac{\lambda^{2}{\sin\beta}^{2}}{W^{2}}\frac{E\varepsilon_{pre}}{1-v} \right)\#\left( 16 \right) \end{aligned}$$

Note that when the pretension becomes zero, Eq. 16 will degenerate into the classical Euler plate instability, and when the pretension is not zero, and the right term of the Eq. 16 dominates the critical stress,

$$\begin{aligned} {\sigma_{\omega}^{cr1}|}_{i=1,j=1}\sim\left\{ \begin{aligned} -\frac{\pi^{2}D}{\lambda^{2}t}, \varepsilon_{pre}=0 \\ -\frac{\lambda^{2}{\sin\beta}^{2}}{W^{2}}\frac{E\varepsilon_{pre}}{1-v}, \varepsilon_{pre}>0 \end{aligned} \right.\#\left( 17 \right) \end{aligned}$$

To determine the primary critical instability stress by Eq. 17, we should know $\lambda$ (the wavelength at which the 1^st^ wrinkling appears for the first time) and $\varepsilon_{pre}$ from experimental results. Here, the pretension strain can be estimated by $\varepsilon_{pre}\sim\frac{\gamma^{cr1}\sqrt{v}\left( 1-v \right)}{2v}$.

To derive the wavelength of wrinkles, we consider the minimization of total energy with wavelength,

$$\begin{aligned} \frac{\partial U}{\partial\lambda}=0\#\left( 18 \right) \end{aligned}$$

Substituting Eq. 8 into Eq. 18, we can obtain,

$$\begin{aligned} i\overline{\lambda}={\frac{i\lambda}{W}\sim\left\{ \frac{2D\pi^{2}\left( 1-v^{2} \right)}{EW^{2}t{\sin\beta}^{2}[-\left( 1+v \right)\varepsilon_{pre}+\left( 1-v \right)\sqrt{{\varepsilon_{pre}}^{2}+\gamma^{2}]}} \right\}}^{\frac{1}{4}}\#\left( 19 \right) \end{aligned}$$

Then, we show the relationship between bending stiffness, pretension and wavelength in Supplementary Fig. 9, where $\xi={[\frac{4D\pi^{2}\left( 1-v^{2} \right)}{EtW^{2}{\sin\beta}^{2}}]}^{\frac{1}{4}}$. Consequently, we can extract the bending stiffness by Eq. 19.

**Supplementary Fig. 9 |** **Theoretical prediction of dimensionless wavelength versus shear strain.** The pretension strain was set as 0, 0.005 and 0.01, respectively.

**Supplementary Fig. 10 |** **The fitting curves to determine the bending stiffness of graphene and MoS_2_.** Error bars represent the standard deviations of measured wavelength data from 5 wrinkles.

**Note 2**

**Origin of bending stiffness fluctuations:**

1. No obvious boundary slippage

In our experiment, we found that there is no boundary slippage (Specific analysis can be found in Section 2), so its effect on the bending stiffness measurements is negligible.

1. Measurement error analysis

The bending stiffness of 2D materials in our experiment can be expressed as

$$D\sim\frac{1}{2\pi^{2}\left( 1-\nu^{2} \right)}\left( \frac{\lambda}{W} \right)^{4}Et W^{2}\sin^{2} \beta\left[ -\left( 1+\nu\right)\varepsilon_{p}+\left( 1-\nu\right)\sqrt{\varepsilon_{p}^{2}+\gamma^{2}} \right]$$

The error of bending stiffness originates from the measurement of *λ*, *β*, *ε_p_*, and *γ*. Then, the error transfer of bending stiffness is given by

$$\left| \frac{\Delta D}{D} \right|=\left| \frac{4\Delta\lambda}{\lambda} \right|+\left| \frac{2\Delta\beta}{\cot\beta} \right|+\frac{\left| \Delta\varepsilon_{p}\left[ -\left( 1+\nu\right)\varepsilon_{p}+\left( 1-\nu\right)\sqrt{\varepsilon_{p}^{2}+\gamma^{2}} \right] \right|+\left| \Delta\gamma\gamma(1-\nu) \right|}{-\left( 1+\nu\right)\varepsilon_{p}\sqrt{\varepsilon_{p}^{2}+\gamma^{2}}+\left( 1-\nu\right)\left( \varepsilon_{p}^{2}+\gamma^{2} \right)}$$

where $\Delta\left[ \cdot\right]$ indicates the variation of parameters, and $\Delta\left[ \cdot\right]/\left[ \cdot\right]$ reflects the relative error. This equation shows the influence of relative error of each measurement quantity on relative error of bending stiffness. Since we obtain the bending stiffness by fitting the function of *λ* with respect to *γ*, the error from *β* and *ε_p_* can be neglected, and only effect of relative error from the half wavelength *λ* is discussed below.

1. Effect of initial corrugations

As atomically thin films, suspended monolayers of 2D materials have been shown to have initial corrugations with undulations in the range of a few nm to a hundred nm. The effect of these corrugations approximate an increase in the equivalent thickness of the 2D materials and therefore leads to an increase in bending stiffness. It should be noted, however, that in SEM experiments, the observation of the initial tiny corrugations is nearly impossible because SEM imaging cannot accurately detect height information. Here, we utilized the same transfer procedures as in this manuscript to transfer monolayer graphene onto the silicon substrates with through-hole arrays (in Supplementary Fig. 11). To avoid the influence of AFM tip on corrugations, we used the non-contact mode AFM to image the height profiles of suspended graphene. As shown in Supplementary Fig. 12, we found that the graphene in the suspended region does have corrugations and these undulations are not significantly oriented. We counted the undulations of graphene in six overhanging regions and found that the undulation heights ranged from a few nanometers to a dozen nanometers. The curves in Supplementary Fig. 12 illustrates the height profile across the line-scanning in each region where the undulations in the three suspended regions ranging from a minimum of approximately 3 nm to a maximum of around 12 nm.

**Supplementary Fig. 11 | Characterization of morphology of monolayer graphene.** **a** Before transfer. **b** Silicon substrate with through-hole arrays.

**Supplementary Fig. 12 | AFM scanning results of suspended monolayer graphene on different holes and the height profile along the line scanning. a-c,** Morphologies of suspended monolayer graphene on three holes where the height profile curves are extracted from the line-scanning along the red lines.

According to theoretical calculation^9^, the effective bending stiffness $D_{eff}$ of the thin film with corrugations $\left\langle A^{2} \right\rangle$ can be written as $D_{eff}/D_{0}=k_{B}TW^{2}/16\pi D_{0}\left\langle A^{2} \right\rangle$, where $k_{B}T$ is the thermal energy, and *D*_0_ is the intrinsic bending stiffness of 2D materials. Correspondingly, if the initial corrugations are 5 nm to 15 nm. the effective bending stiffness $D_{eff}$ is 5 eV to 47 eV, which agrees well to the range of experimental measurement. Additionally, the amplitude the initial fluctuation is restrained by the prestrain, given by $\left\langle A^{2} \right\rangle=\frac{k_{B}T}{4\pi E\varepsilon_{pre}}\ln\left( 1+\frac{EW^{2}\varepsilon_{pre}}{4\pi^{2}D_{0}} \right)$. Due to the random prestrains, the initial fluctuations of our sample are not completely consistent, leading to different initial fluctuations in Supplementary Fig. 10.

1. Possible effect of irradiations

According to the references, ion beam irradiation will have a significant effect on the mechanical properties of 2D materials^10^. In our experiments, we selected samples with regular boundaries without the need for ion beam processing, so the effect of ion irradiation can be neglected. On the other hand, high-voltage electron microscopy, such as transmission electron microscopy electron beams, can damage 2D materials and cause defects, as well as affect bending properties. Here, we used a SEM voltage of 10kV, so 2D materials will not be damaged by the bombardment of high-energy electron beam streams. Besides, to avoid large amounts of amorphous carbon deposit during the test, we used the plasma treatment to clean the electron microscope chamber before each experiment, thus minimizing the effect of amorphous carbon on the measurements. However, we also realized that, when the sample is exposed to prolonged periods within the electron microscope, amorphous carbon will accumulate, leading to increased values in subsequent bending stiffness measurements.

1. Effect of multiple measurements

In Supplementary Fig. 13**a**, we present the unprocessed force-displacement curves of the same sample under different loading-unloading cycles. It is noteworthy that the forces align closely during both loadings, implying that the material's stress-strain response remains largely consistent with the increasing number of measurements. Note that, as the 3^rd^ loading surpasses the strength of monolayer graphene, an abrupt decrease in force curve is evident in loading stage. This decline signifies shear damage to the material. Besides, the SEM images captured in two cyclic loadings are shown in Supplementary Fig. 13**b**, both of which indicate the wrinkle-splitting phenomena. To investigate the effect of multiple measurements on wrinkling patterns, in Supplementary Fig. 14, we present the wrinkling behavior (1^st^ instability) observed in the same sample subjected to three loading-unloading cycles. At initial strains (<1%), the differences in wavelength are discernible among the three loadings. These findings suggest that, following every loading, there is a shift in the prestrains of the sample. This phenomenon is attributed to the fact that unloading does not fully restore the initial state attained after the last loading, consequently giving rise to residual strain. At larger shear strains, the relationship of wavelengths under the three loadings observed as $\bar{\lambda}_{1}<\bar{\lambda}_{2}\bar{<\lambda}_{3}$, signifies that, as the measurements increase, the bending stiffness of the sample also experiences an incremental rise.

**Supplementary Fig. 13 | Comparison of cyclic loading-unloading behaviors of monolayer graphene. a** The force-displacement curves of the same sample during three cyclic loadings. **b** The wrinkling splitting occurring in second and third cyclic loadings. Scale bars: **a**, **b** 2 μm.

**Supplementary Fig. 14 | Wrinkling behaviors of Monolayer graphene in different cyclic loadings.** Solid balls indicate the normalized wrinkling wavelengths measured in the same sample during three cyclic loadings. The curves show the theoretical normalized wrinkling wavelengths versus shear strains with different bending stiffness. Error bars represent the standard deviations of measured wavelength data from 5 wrinkles.

In our previous analysis, we illustrated the significant impact of the initial corrugations on bending stiffness. The divergence in bending stiffness under various loadings arises, on one hand, from alterations in the initial corrugations after each loading cycle. On the other hand, as such tests were mostly conducted under an electron microscope, prolonged exposure may result in the formation of amorphous carbon deposits on the material surface. These deposits may modify the initial corrugations and thus increase effective thickness of the sample. These factors also contribute to an upward trend in the bending stiffness with increased numbers of tests.

**Note 3**

**Secondary (2^nd^) instability:**

For the secondary instability problem, it corresponds to that there are two states with different wavenumbers at the same stress state, such as the doubling wavenumber seen in the experiment should be described as,

$$\begin{aligned} {\sigma_{\omega}|}_{i_{1},j=1}={\sigma_{\omega}|}_{i_{2},j=1}\#\left( 20 \right) \end{aligned}$$

Here, we define the scale factor between the two stresses as$\eta=-\sigma_{\psi}{/\sigma}_{\omega}$, then we can obtain from Eq. 11,

$$\begin{aligned} \frac{\left( \frac{{i_{1}}^{2}}{\lambda^{2}}+\frac{{\sin\beta}^{2}}{W^{2}} \right)^{2}}{\left( \frac{{i_{1}}^{2}}{\lambda^{2}}+\frac{\eta{\sin\beta}^{2}}{W^{2}} \right)}=\frac{\left( \frac{{i_{2}}^{2}}{\lambda^{2}}+\frac{{\sin\beta}^{2}}{W^{2}} \right)^{2}}{\left( \frac{{i_{2}}^{2}}{\lambda^{2}}+\frac{\eta{\sin\beta}^{2}}{W^{2}} \right)}\#\left( 21 \right) \end{aligned}$$

By solving Eq. 21, the critical wavelength of the secondary instability is related to the stress ratio coefficient and material geometry,

$$\begin{aligned} \lambda=\frac{\sqrt{\left( 2\eta+1 \right)[-\left( {i_{1}}^{2}+{i_{2}}^{2} \right)\eta+\sqrt{\left( {i_{1}}^{2}+{i_{2}}^{2} \right)^{2}\eta^{2}+\left( 4+8\eta\right){i_{1}}^{2}{i_{2}}^{2}}]}}{2\eta+1}\frac{W}{\sin\beta}\#\left( 22 \right) \end{aligned}$$

We consider $i_{1}=1, i_{2}=2$, that is, the second instability corresponds to the period-halving bifurcation of wavelength, so we get that,

$$\begin{aligned} \overline{\lambda^{cr2}}=\frac{\lambda}{W}=\frac{\sqrt{\left( 2\eta+1 \right)(-5\eta+\sqrt{25\eta^{2}+32\eta+16})}}{(2\eta+1)sin\beta}\#\left( 23 \right) \end{aligned}$$

In practice, we can use these equations to derive secondary critical shear strain $\gamma^{cr2}$: $Eqs. 13\to\varepsilon_{pre}$; $Eqs. 17 \& 4\to\eta$; $Eqs. 23\to\overline{\lambda^{cr2}}$; $Eqs. 19\to\gamma^{cr2}$. Basically, $\gamma^{cr2}$ is related to $\gamma^{cr1}$, as shown in Supplementary Fig. 15. Such procedure is similar to period fission analysis in thin film wrinkling^11^.

Furthermore, we can obtain the critical stress for 2^nd^ instability as,

$$\begin{aligned} \overline{\sigma_{\omega}^{cr2}}=\frac{W^{2}t\sigma_{\omega}^{cr2}}{\pi^{2}D{\sin\beta}^{2}}=\left( 1+{\overline{\lambda^{cr2}}}^{2} \right)^{2}\left( 1-\eta{\overline{\lambda^{cr2}}}^{2} \right)^{-1}\#\left( 24 \right) \end{aligned}$$

**Supplementary Fig. 15 | Theoretical prediction of** **dimensionless secondary critical wavelength versus primary critical shear strain.** Here, dimensionless secondary critical wavelengths were set as 0.1, 0.3 and 0.6, respectively.

**Supplementary Fig. 16 | Schematics of instability-recovery process. a-c,** Schematics of wrinkling splitting process. **d-f,** Schematics of smoothing process.

**Wrinkling splitting during 1^st^-to-2^nd^ wrinkling:**

According to Section 3, we know that the critical stresses for 1^st^ and 2^nd^ instability ($\sigma_{\omega}^{cr1}$ and $\sigma_{\omega}^{cr2}$) can be obtained by Eqs. S17 and S24. During loading stage, when the compressive stress increased above $\sigma_{\omega}^{cr2}$, the wrinklon would appear as a result of the bifurcation (2^nd^ instability initiated). Therefore, the wrinkling splitting can be equivalent to the growth of wrinklon. According to previous wrinklon model, we know that the length of wrinklon can be related to wavelength and external tension^12^,

$$\begin{aligned} L_{wrinklon}\sim{\lambda_{wrinklon}}^{2}T^{\frac{1}{2}}\sim\lambda^{2}{\sigma_{\psi}}^{\frac{1}{2}}\#\left( 25 \right) \end{aligned}$$

To simplify the analysis, the effects of pretension are ignored. We suppose $\lambda_{wrinklon}\sim\gamma^{-\frac{1}{4}}$, $T\sim\sigma_{\psi}\sim\gamma$ through Eqs. 19 and 4, then $L_{wrinklon}$ should be constant. However, we observed that the length of the wrinklon was increasing, indicating that in the experiment, $\lambda_{wrinklon}$ is no longer obeys $\gamma^{-\frac{1}{4}}$ after when the 2^nd^ instability initiated. Here we speculate that $\lambda_{wrinklon}$ would stay constant during the splitting process of 1^st^ wrinkle, so $L_{wrinklon}$would increase following Eq. 25 until the splitting completed.

**Smoothing process during 2^nd^-to-flat wrinkling:**

Normally, the 2^nd^ wrinkles will merge along the opposite path of bifurcation during the unloading stage, that is, when the compressive stress falls below $\sigma_{\omega}^{cr2}$, the 2^nd^ wrinkles should merge into 1^st^ wrinkles. However, in our experiment, we found the 2^nd^ wrinkles prefer decreased their amplitude instead of merged to increase the wavelength. We infer the reason is that the 2D material has relieved the compressive stress to a very low level (${\ll\sigma}_{\omega}^{cr2}$) when the bifurcation completed during loading stage^13^. Hence, after the unloading started, the compressive stress would not pass through $\sigma_{\omega}^{cr2}$, so the merging of wrinkles would hardly occur during unloading stage. In fact, previous researchers have found that monolayer graphene has an ultra-high stress or strain release rate^14^. So we may attribute the symmetry breaking in instability and recovery trajectories to the fast relieve of compressive stress. To further interpret our conjecture by stress analysis, we will discuss the question further with the MD simulation in next section.

**Simulation settings**

To simulate the wrinkle evolution and binary buckling process, the upper edge was moved at a constant velocity of 5 m/s with a thermal relaxation time of 2 ps. During shearing, the whole system was exposed to a constant temperature of 10 K via an NVT ensemble. When the shear strain reaches a critical value ($\gamma= 4.83\%$), the upper edge was applied to a constant velocity in the opposite direction to simulate the unloading process. The shear stress was defined as the mean atomic shear stress of whole system divided by atomic volume. To obtain the atomic stress field, we performed molecular simulations using CG algorithm. Before shearing process, each atom except fixed edge in monolayer graphene was randomly assigned an out-of-plane displacement of less than 0.1 Å. The shearing process was then simulated by loading step by step. In each step, the upper edge was move at constant displacement of 0.1 Å, and the total system was then relaxed through energy minimization with energy convergence condition of 1.0 × 10^-12^. The simulation stopped when the shear strain reaches $\gamma= 5.0\%$. The maximum and minimum principal stresses were calculated by the atomic virial stress, and direction of maximum principal stress was defined as the its angle with *x*-direction.

**
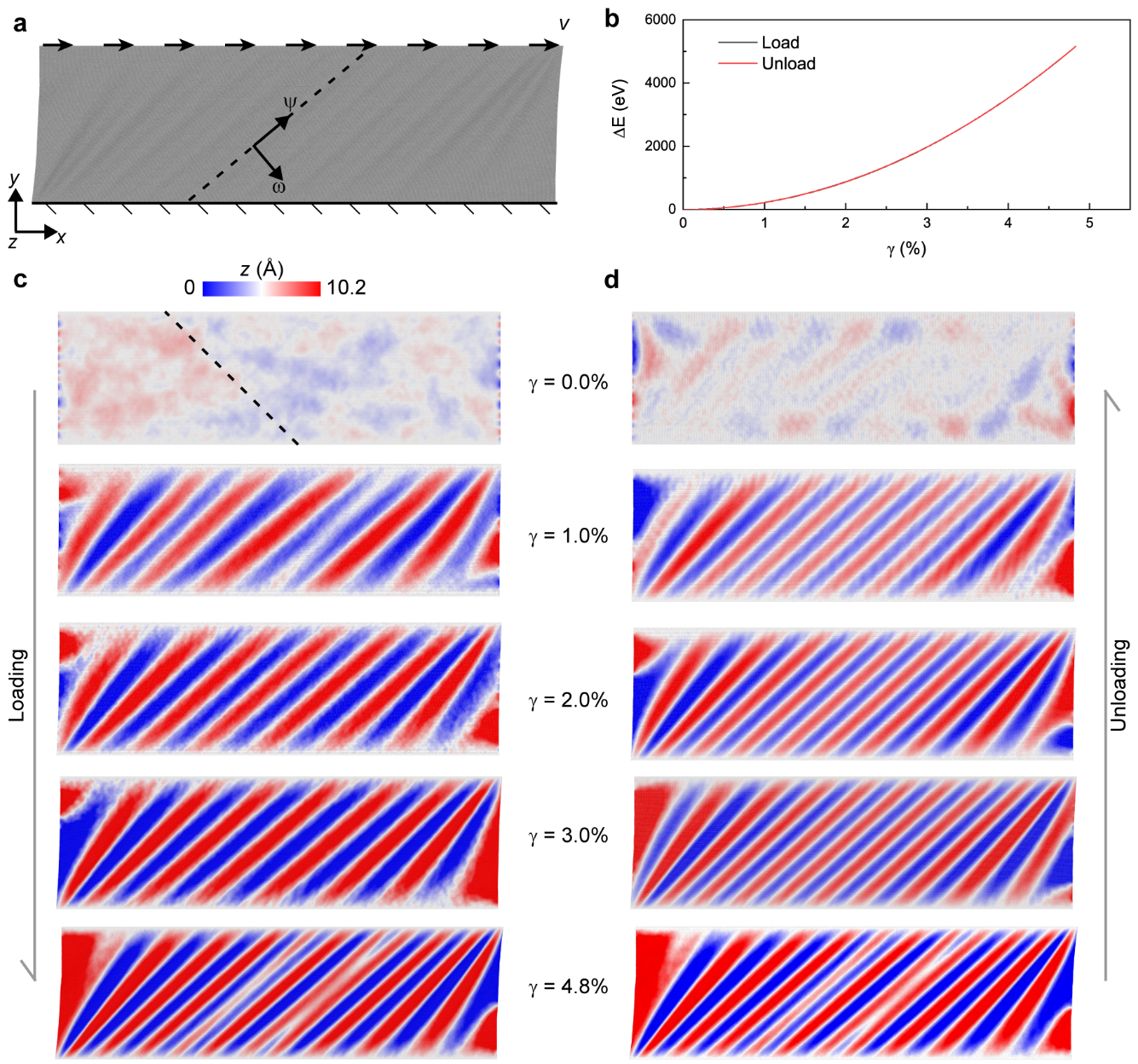
**

**Supplementary Fig. 17 | Reversible instability of monolayer graphene in MD simulation.** **a** Schematic diagram of a shear-wrinkling simulation model. **b** Variation of system potential energy during loading and unloading. Although the instability paths are asymmetric, the curves of system potential energy are almost overlapped during loading and unloading process. Wrinkle evolution during loading **c** and unloading **d** process. These atomic configurations show the asymmetry in wrinkle amplitude and wavelength. The monolayer graphene can recover its initial configuration after a loading-unloading cycle.

**
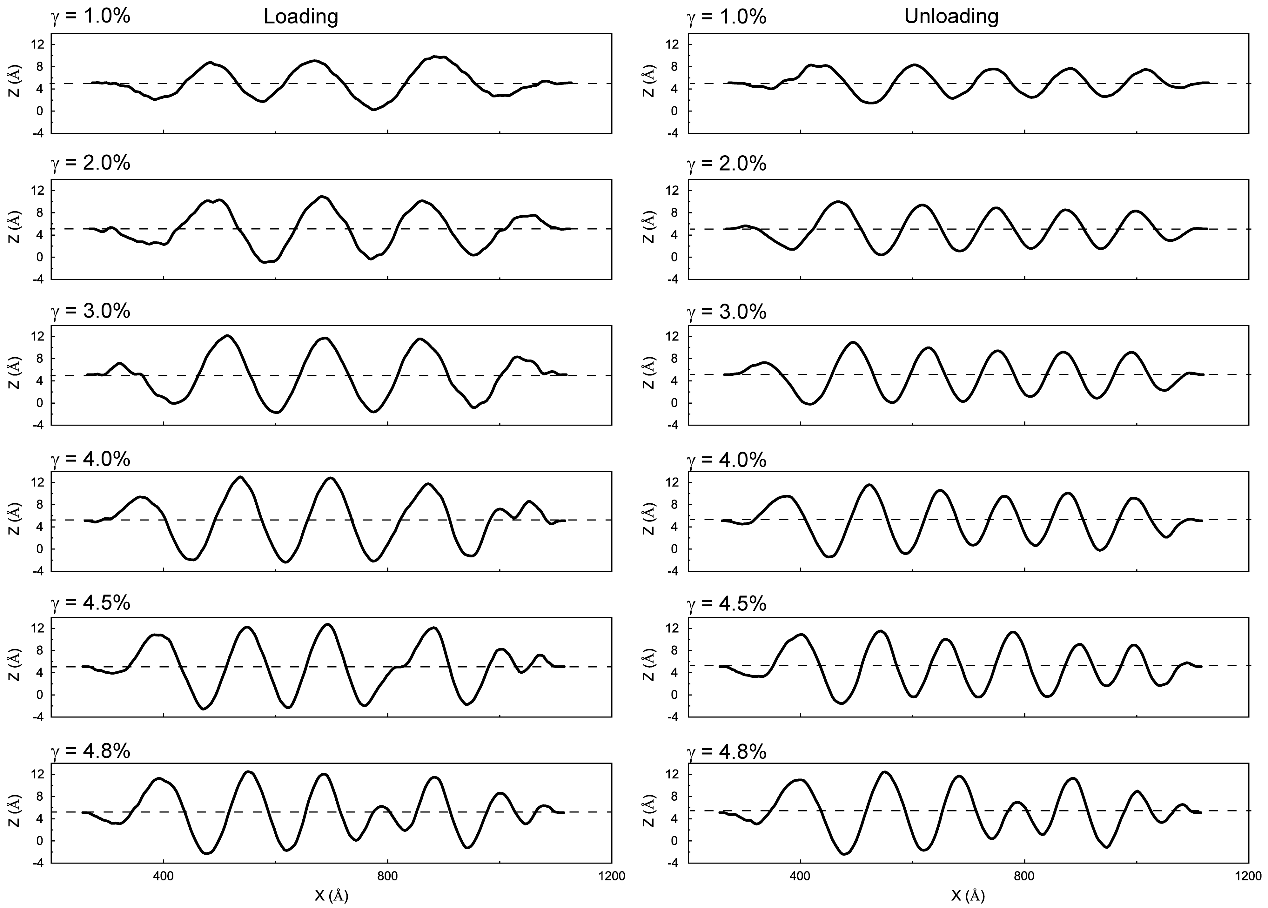
**

**Supplementary Fig. 18 | Evolution of wrinkle profiles in MD simulation during loading-unloading.** The curves are extracted from the cross section indicated in Supplementary Fig. 17**b**, which exhibits wrinkling splitting and merging process.

**
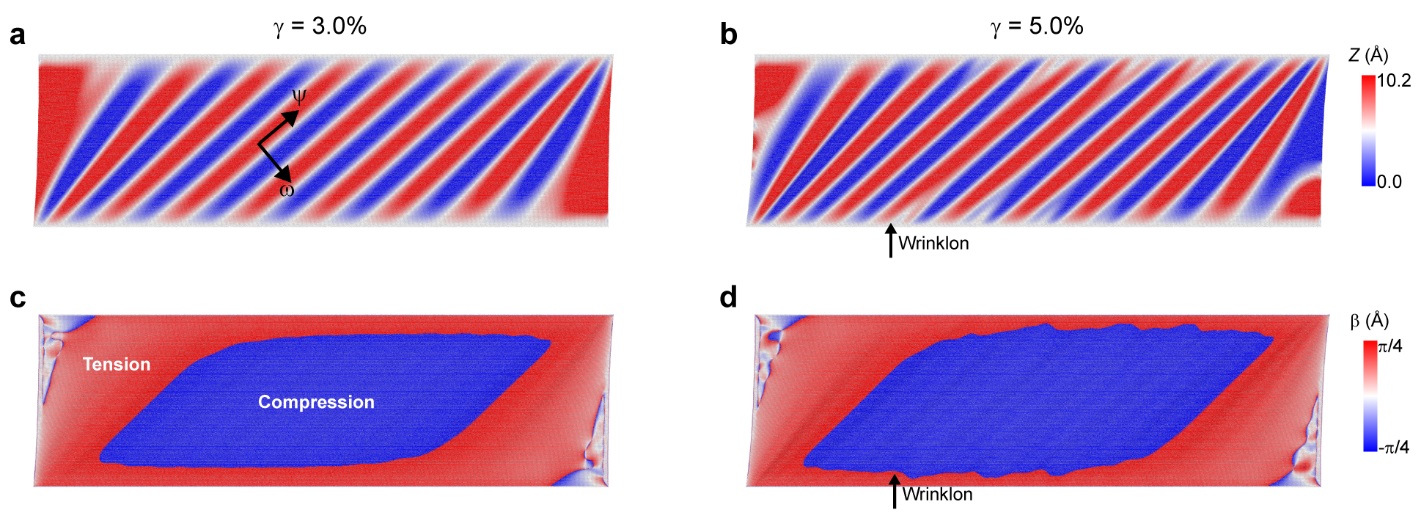
**

**Supplementary Fig. 19 | Morphology and stress evolution of graphene during loading process.** Buckling configurations under different shear strain$\gamma= 3.0\%$ **a** and $\gamma= 5.0\%$ **b**. Distribution of principal stress direction in a monolayer graphene at shear strain of $\gamma= 3.0\%$ **c** and $\gamma= 5.0\%$ **d**. $\beta> 0$ (red region) indicates the maximum principal stress is along the wrinkle (*ψ*) direction, and wrinkle is stretched. $\beta< 0$ (blue region) indicates the minimum principal stress is perpendicular to the wrinkle (*ω*) direction, and wrinkle is compressed.

**Supplementary References**

1. Schneider GF, Calado VE, Zandbergen H, Vandersypen LM, Dekker C. Wedging transfer of nanostructures. *Nano Lett.* **10**, 1912-1916 (2010).

2. Hou Y, et al. Preparation of twisted bilayer graphene via the wetting transfer method. *ACS Appl. Mater. Interfaces* **12**, 40958-40967 (2020).

3. Kitt AL, Qi Z, Rémi S, Park HS, Swan AK, Goldberg BB. How graphene slides: measurement and theory of strain-dependent frictional forces between graphene and SiO_2_. *Nano Lett.* **13**, 2605-2610 (2013).

4. Dai Z, et al. Interface-governed deformation of nanobubbles and nanotents formed by two-dimensional materials. *Phys. Rev. Lett.* **121**, 266101 (2018).

5. Wang G, et al. Measuring interlayer shear stress in bilayer graphene. *Phys. Rev. Lett.* **119**, 036101 (2017).

6. Wang G, Gao E, Dai Z, Liu L, Xu Z, Zhang Z. Degradation and recovery of graphene/polymer interfaces under cyclic mechanical loading. *Com. Sci. Tech.* **149**, 220-227 (2017).

7. Wong W, Pellegrino S. Wrinkled membranes II: analytical models. *J. Mech. Mater. Struct.* **1**, 27-61 (2006).

8. Wang C, Tan H, Lan L, Li L. Mode jumping analysis of thin film secondary wrinkling. *Int. J. Mech. Sci.* **104**, 138-146 (2015).

9. Ahmadpoor F, Wang P, Huang R, Sharma P. Thermal fluctuations and effective bending stiffness of elastic thin sheets and graphene: A nonlinear analysis. *J. Mech. Phys. Solids* **107**, 294-319 (2017).

10. López-Polín G, et al. Increasing the elastic modulus of graphene by controlled defect creation. *Nat. Phys.* **11**, 26-31 (2015).

11. Davidovitch B. Period fissioning and other instabilities of stressed elastic membranes. *Phys. Rev. E* **80**, 025202 (2009).

12. Vandeparre H, et al. Wrinkling hierarchy in constrained thin sheets from suspended graphene to curtains. *Phys. Rev. Lett.* **106**, 224301 (2011).

13. Schroll RD, Katifori E, Davidovitch B. Elastic building blocks for confined sheets. *Phys. Rev. Lett.* **106**, 074301 (2011).

14. Belyaeva LA, Jiang L, Soleimani A, Methorst J, Risselada HJ, Schneider GF. Liquids relax and unify strain in graphene. *Nat. Commun.* **11**, 898 (2020).
