# Supplementary material for: Tuning instability in suspended monolayer 2D materials
Source: Nat Commun. 2024 May 13;15:4033. doi: 10.1038/s41467-024-48345-7 (PMC11091077; doi:10.1038/s41467-024-48345-7)
Supplement: Supplementary file 3 — Description of Additional Supplementary Information [file 41467_2024_48345_MOESM3_ESM.pdf]

Description of Additional Supplementary Information

Supplementary Movie 1: Shear-induced instability of monolayer graphene.

Supplementary Movie 2: Shear-induced instability of monolayer MoS2.

Supplementary Movie 3: Shear loading-unloading of monolayer graphene with small strains.

Supplementary Movie 4: MD simulation (height map).

Supplementary Movie 5: MD simulation (stress map).
